# The Phubbing Scale (PS-8) in the Portuguese population: psychometric properties

**DOI:** 10.1186/s41155-022-00209-z

**Published:** 2022-03-16

**Authors:** F. Javier García-Castro, Ana Maria Abreu, Belén Rando, María J. Blanca

**Affiliations:** 1 Department of Psychobiology and Behavioral Sciences Methodology, University of Malaga, Malaga, Spain; 2 Universidade Católica Portuguesa, Center for Interdisciplinary Research in Health, (CIIS), Institute of Health Sciences, Lisbon, Portugal; 3 Institute of Social and Political Sciences, University of Lisbon, Lisbon, Portugal; 4 Centre for Public Administration and Public Policies (CAPP), Lisbon, Portugal; 5 Facultad de Psicología, Campus Universitario de Teatinos, 29071 s/n. Málaga, Spain

**Keywords:** Communication disturbance, Phone obsession, Internet addiction, Problematic mobile phone use, Confirmatory factor analysis, Psychometric properties

## Abstract

Phubbing is defined as ignoring other individuals by using a mobile phone during a face-to-face conversation. The Phubbing Scale (PS) was developed to assess this practice. In this study, we analyze the psychometric properties of the 8-item version of the PS (PS-8) in the Portuguese population, providing validity evidence based on internal structure and on relationships with other variables, and examining item properties, reliability, and measurement invariance across gender. Participants were 391 Portuguese adults (132 men, 259 women) who completed a battery of questionnaires. Confirmatory factor analysis yielded satisfactory goodness-of-fit indices for the two-factor structure (communication disturbance and phone obsession), which was invariant across gender. Item homogeneity and reliability of factor scores (McDonald’s omega) were satisfactory. Validity evidence based on relationships with other variables was provided by positive associations with time spent on the Internet on weekdays and at the weekend, time spent on social networking sites, number of social networks used, Internet addiction, problematic mobile phone use, Facebook intrusion, fear of missing out, and depression. These associations show the addictive component of phubbing and its relationship with mental health. The PS-8 is a short and easy-to-administer scale with adequate psychometric properties for measuring phubbing in the Portuguese population.

## Introduction

Mobile phones have become an inseparable part of daily life (Chotpitayasunondh & Douglas, 2016). They now have the properties of pocket computers and enable people to communicate and interact with others regardless of time and place (Gomes et al., 2021; Karadağ et al., 2015). Despite their benefits, however, mobile phones also have potential negative effects. For example, although they can facilitate social interactions, they may also interfere with relationships when used in the presence of others (Chotpitayasunondh & Douglas, 2016, 2018; Ivanova et al., 2020). This phenomenon is known as *phubbing*, a combination of the words “phone” and “snubbing”, and it is defined as ignoring other individuals by using a mobile phone during a face-to-face conversation (Chotpitayasunondh & Douglas, 2016; Karadağ et al., 2015).

Recent years have seen a growing interest in the study of phubbing (Chotpitayasunondh & Douglas, 2016; Fang et al., 2020). In order to assess this behavior, Karadağ et al. (2015) developed the 10-item Phubbing Scale (PS). They identified two factors, each with five items: communication disturbance, referring to how individuals disturb their communication by dealing with their mobile phones in a face-to-face communication environment, and phone obsession, reflecting the extent to which individuals need their mobile phone in environments lacking face-to-face communication. Karadağ et al. (2015) found that scores on these two factors are positively correlated with several addictions, namely to mobile phones, SMS, the Internet, social networks, and gaming. These relationships between phubbing and addictions have been consistently reported (e.g., Al-Saggaf & O’Donnell, 2019; Błachnio & Przepiorka, 2019; Blanca & Bendayan, 2018; Davey et al., 2018; Fang et al., 2020; Ivanova et al., 2020; Yam & Kumcağız, 2020), with phubbing being considered the outcome of combined virtual addictions (Karadağ et al., 2015).

Research has also shown that the practice of phubbing is related to being permanently online and connected, and in this context, to higher levels of Facebook intrusion and fear of missing out (Al-Saggaf & O’Donnell, 2019; Błachnio & Przepiorka, 2019; Blanca & Bendayan, 2018; Davey et al., 2018; Fang et al., 2020; Schneider & Hitzfeld, 2019; Yam & Kumcağız, 2020). In addition, it is positively associated with boredom, neuroticism, and conscientiousness, and negatively associated with self-esteem and self-control (Al-Saggaf et al., 2019; Al-Saggaf & O’Donnell, 2019; Davey et al., 2018; Erzen et al., 2021; Ivanova et al., 2020; Yam & Kumcağız, 2020). The practice of phubbing may also have a negative impact on health, in the form of increased loneliness, depression, and distress, and decreased well-being, life satisfaction, and flourishing (Al-Saggaf & O’Donnell, 2019; Błachnio & Przepiorka, 2019; Davey et al., 2018; Ergün et al., 2020; Ivanova et al., 2020).

Although the PS has been used in many studies to assess phubbing (e.g., Błachnio & Przepiorka, 2019; Blanca & Bendayan, 2018; Davey et al., 2018; Ergün et al., 2020; Erzen et al., 2021; Gomes et al., 2021; Ivanova et al., 2020; Parmaksiz, 2019), instrumental studies analyzing its psychometric properties are scarce. Blanca and Bendayan (2018) developed the Spanish version of the PS, providing evidence of the aforementioned two-factor structure and validity evidence based on positive associations with measures of Internet addiction, Facebook intrusion, and fear of missing out. More recently, Błachnio et al. (2021) analyzed the factor structure and measurement invariance of the PS across 20 countries. They tested one-factor and two-factor models, both including the 10 original items. However, these structures were not invariant across all countries. The authors then developed a refined scale consisting of just eight items (hereinafter, the PS-8), and provided evidence of its two-factor structure (four items per factor), which was invariant across the 20 countries. They also provided evidence of measurement invariance across gender. Although Portugal was one of the countries included in this study, further research into the psychometric properties of the PS-8 is warranted in order to provide more validity evidence about its use in the Portuguese population and validity evidence based on relationships with other variables, the latter being an aspect that was not addressed in the study by Błachnio et al. (2021). In this context, it is particularly relevant to provide new evidence of the measurement invariance across gender to demonstrate whether the instrument has the same factor pattern and whether items reflect the same latent construct for men and women. It should be noted that Água et al. (2019) developed a Portuguese adaptation of another instrument that assesses phubbing, namely the Partner Phubbing Scale (Roberts & David, 2016). However, this scale assesses the practice of phubbing solely within the context of a romantic relationship.

Given that the PS-8 allows for comparisons across countries and hence is potentially applicable in future cross-cultural research (Błachnio et al., 2021), the goal here was to extend knowledge about its use in the Portuguese population. Specifically, we aimed to provide validity evidence based on the internal structure of the PS-8 to examine measurement invariance across gender, to analyze the reliability of test scores, and item homogeneity, and to provide validity evidence based on relationships with other relevant variables, namely, time spent on the Internet on weekdays and at the weekend, time spent on social networking sites, number of social networks used, Internet addiction, problematic mobile phone use, Facebook intrusion, fear of missing out, and depression. We expected to obtain satisfactory fit indices for the correlated two-factor structure for the PS-8 (communication disturbance and phone obsession), with measurement invariance across gender, satisfactory reliability coefficients for scores on both factors, adequate item homogeneity, and positive relationships of both factors with time spent on the Internet on weekdays and at the weekend, time spent on social networking sites, number of social networks used, Internet addiction, problematic mobile phone use, Facebook intrusion, fear of missing out, and depression.

## Methods

### Participants

The sample comprised 391 Portuguese adults (132 men, 259 women) ranging in age from 18 to 52 years (*M* = 26.16, SD = 8.72). To be eligible, participants had to be 18 years or older, have Portuguese nationality and residence, and be a mobile phone user. All participants who did not meet these inclusion criteria would be excluded from the analysis. The sociodemographic characteristics of participants are shown in Table 1.

**Table 1:** Sociodemographic characteristics of the sample (*N* = 391)

Variable	Percentage
Gender	
Men	66.24
Women	33.76
Marital status	
Single	76.98
Married	20.46
Divorced	2.56
Level of education	
Primary	0.26
Secondary	48.08
University	51.66
Employment status	
Student	55.50
Employed	30.43
Student and employed	12.02
Unemployed/retired	2.05

### Instruments

#### Phubbing

The Phubbing Scale (PS; Karadağ et al., 2015) comprises 10 items, each rated on a 5-point Likert-type scale (1 = never; 5 = always) and distributed across two factors (5 items each): communication disturbance, which measures how individuals disturb their communication by dealing with their mobile phones in a face-to-face communication environment; and phone obsession, reflecting the extent to which individuals need their mobile phone in environments lacking face-to-face communication. For the present study, we used the Portuguese version of the refined 8-item scale (PS-8) proposed by Błachnio et al. (2021), in which each factor comprises four items (see Table 2). Scores on both factors range from 4 to 20.

**Table 2:** Means (M), standard deviations (SD), skewness (S), and kurtosis (K) for PS-8 items, its two factors, and the other study variables (*N* = 391)

Variables	*M*	*SD*	*S*	*K*
1. My eyes start wandering on my phone when I’m together with others [Quando estou com outras pessoas, costumo estar de olho no telemóvel]	2.74	0.92	0.38	− 0.07
2. I am busy with my mobile phone when I’m with my friends [Quando estou com os meus amigos/as, costumo estar ocupado com o meu telemóvel]	2.11	0.71	0.41	0.50
3. People complain about me dealing with my mobile phone [As pessoas queixam-se por eu estar no telemóvel]	1.66	0.83	1.26	1.30
4. I’m busy with my mobile phone when I’m with my family [Estou ocupado/a com o meu telemóvel quando estou com familiars]	2.27	0.86	0.42	− 0.19
5. My phone is within my reach [O meu telemóvel está sempre ao meu alcance]	3.88	1.07	− 0.65	− 0.52
6. When I wake up in the morning, I first check the messages on my phone [Quando acordo de manhã, o primeiro que faço é verificar as mensagens no meu telemóvel]	3.20	1.41	− 0.16	− 1.29
7. I feel incomplete without my mobile phone [Sinto-me incompleto/a sem o meu telemóvel]	2.87	1.28	0.08	− 1.09
8. My mobile phone use increases day by day [De dia para dia o meu uso do telemóvel aumenta]	2.18	1.01	0.59	− 0.27
Communication Disturbance	8.78	2.67	0.61	0.67
Phone Obsession	12.13	3.55	− 0.06	− 0.65
Time spent on the Internet on weekdays	6.30	3.25	0.05	− 1.18
Time spent on the Internet at the weekend	5.66	3.38	0.23	− 1.10
Time spent on social networking sites	3.38	1.27	0.24	− 0.37
Number of social networks used	4.91	1.59	0.18	− 0.20
Internet addiction	13.57	4.59	0.48	− 0.36
Problematic mobile phone use	19.47	4.84	0.44	− 0.10
Facebook intrusion	17.44	7.68	1.06	0.91
Fear of missing out	21.37	6.98	0.52	0.02
Depression	9.83	5.96	0.70	− 0.04

#### Time spent on the Internet on weekdays

To assess the number of daily hours of Internet use on weekdays, participants were asked: ‘Approximately, how many hours per day do you spend on the Internet on weekdays?’. Responses were given on a 12-point scale (0 = less than an hour; 11 = more than 10 h).

#### Time spent on the Internet at the weekend

To assess the number of daily hours of Internet use at the weekend, participants were asked: ‘Approximately, how many hours per day do you spend on the Internet at the weekend?’. Responses were given on the same 12-point scale (0 = less than an hour; 11 = more than 10 h).

#### Time spent on social networking sites

To assess the frequency of their social network use, participants were asked: ‘Approximately, how much time do you spend on social networking sites?’. Responses were given on a 6-point scale (1 = I rarely use social networks; 2 = less than 1 h per day; 3 = 1–3 h per day; 4 = 3–5 h per day; 5 = 5–8 h per day; 6 = more than 8 h per day).

#### Number of social networks used

Participants were asked to indicate (yes/no) whether they used the following social networks: Facebook, Instagram, Twitter, LinkedIn, WhatsApp, Snapchat, Pinterest, YouTube, Others. The total number of social networks used was obtained by summing their affirmative responses (range 0 to 9).

#### Internet addiction

This was assessed using the Internet Addiction Scale (IAS; Karadağ et al., 2015), which comprises six items, each rated on a 5-point Likert-type scale (1 = never; 5 = always). The total score therefore ranges from 6 to 30, and higher scores indicate a higher level of addiction to the Internet. Cronbach’s alpha coefficient in the present sample was .78.

#### Problematic mobile phone use

Participants completed the Adapted Mobile Phone Use Habits scale (AMPUH; Smetaniuk, 2014), which assesses behavioral characteristics associated with addictive mobile phone use. The AMPUH comprises 10 items, each rated on a 5-point Likert-type scale (1 = never; 5 = always) and yields a total score ranging from 10 to 50. Higher scores indicate more problematic mobile phone use. Cronbach’s alpha coefficient in the present sample was .68.

#### Facebook intrusion

We administered the Facebook Intrusion Questionnaire (FIQ; Elphinston & Noller, 2011), which assesses excessive attachment to Facebook and how this interferes with daily activities and relationship functioning. The FIQ comprises eight items, each rated on a 7-point Likert-type scale (1 = strongly disagree; 7 = strongly agree), and hence the total score ranges from 8 to 56. Higher scores indicate higher levels of Facebook intrusion. Cronbach’s alpha coefficient in the present sample was .82.

#### Fear of missing out

Participants completed the Fear of Missing Out scale (FoMOs; Przybylski et al., 2013), which assess worries about being excluded from rewarding experiences that others are having. The FoMOs comprises 10 items, each rated on a 5-point Likert-type scale (1 = not at all true of me; 5 = extremely true of me) and yields a total score ranging from 10 to 50. Higher scores indicate higher levels of fear of missing out. Cronbach’s alpha coefficient in the present sample was .83.

#### Depression

The short form of the Center for Epidemiologic Studies Depression Scale (CESD; Andresen et al., 1994; Radloff, 1977) was administered. The CESD assesses depressive symptoms experienced during the last week by means of 10 items, each rated on a 4-point Likert-type scale (0 = rarely or none of the time; 3 = most or all the time). The total score ranges from 0 to 30, and higher scores indicate higher levels of depression. Cronbach’s alpha coefficient in the present sample was .84.

### Procedure

Participants completed an online survey, which was distributed through an ad hoc blog and major social networks in Portugal (LinkedIn, Facebook, and WhatsApp), following a snowball sampling strategy. The link to the ad hoc blog, along with an explanation of the study and anonymity and confidentiality guidelines, was also shared with several colleagues and students who were asked to share the link on their social networks and to require further respondents to reshare the link and so on. Before completing the online survey, participants were informed about the purpose of the study, and that all responses would remain anonymous and would be used for research purposes only. Then the participants provided informed consent to access the survey, indicating that they were over 18 years of age, had Portuguese nationality and residence, and were mobile phone users. The time required to complete the survey was around 15–20 min. No potential participants were excluded, and there were no missing data as all the questions needed to be answered in order to submit the survey. The study was carried out in agreement with the Declaration of Helsinki, and it was approved by the Research Ethics Committee of the University of Malaga.

### Data analysis

We began by conducting a descriptive analysis, calculating means, standard deviations, and skewness and kurtosis coefficients for PS-8 items and the other study variables. Next, and in order to obtain validity evidence based on the internal structure of the PS-8, we performed a confirmatory factor analysis (CFA) using EQS 6.4 software (Bentler, 2006). We tested the model proposed by Błachnio et al. (2021), composed of two correlated latent factors: communication disturbance (items 1, 2, 3, and 4) and phone obsession (items 5, 6, 7, and 8). The CFA was based on the polychoric correlation matrix and used maximum likelihood and robust estimation methods. Model fit was assessed using the Satorra-Bentler chi-square statistic (*χ*^2^_S-B_) and the following fit indices: the comparative fit index (CFI; Bentler, 1990), the non-normed fit index (NNFI; Bentler & Bonett, 1980), and the root mean square error of approximation (RMSEA; Browne & Cudeck, 1993; Steiger, 2000). Values of the CFI and the NNFI above .95 are considered to indicate a good fit (Hu & Bentler, 1999), whereas for the RMSEA, values between .06 and .08 indicate a reasonable fit (Browne & Cudeck, 1993), and those below .06 a good fit (Hu & Bentler, 1999). We also tested configural and metric invariance across gender which encompassed a hierarchical set of steps. The process began with the determination of the baseline model for men and women, separately, obtaining their respective fit indices. Once the fit indices supported the correlated two-factor model in both groups, configural invariance was analyzed. Configural invariance was tested by constraining the factorial structure to be the same across gender in order to test whether the same number of factors and factor-loading pattern was the same for men and woman, with no equality constraints imposed on the parameters (Byrne, 2006). Configural invariance was tested by assessing the model fit, providing the baseline value against which the metric invariance was compared. Metric invariance was then tested, firstly, by introducing equality constraints on factor loadings and, secondly, on factor loadings and covariance between factors across gender in order to test whether items reflect the same latent construct for men and women. This invariance was assessed by comparing its CFI with the CFI of the configural model. Equality constraints are tenable when the decrease in CFI in these models is less than or equal to .01 in relation to the configural model (Byrne, 2008; Byrne & Stewart, 2006; Cheung & Rensvold, 2002).

Item properties were then analyzed by computing corrected item-total correlation coefficients, with values above .30 being considered satisfactory. To test the reliability of items, we computed McDonald’s omega coefficients. Values above .70 are generally considered as acceptable (Dunn et al., 2014; Viladrich et al., 2017).

Finally, we obtained validity evidence based on relationships with other variables by calculating Pearson correlation coefficients between scores on the PS-8 and scores on the other study variables (i.e., time spent on the Internet on weekdays and at the weekend, time spent on social networking sites, number of social networks used, Internet addiction, problematic mobile phone use, Facebook intrusion, fear of missing out, and depression). Coefficients were interpreted according to Cohen’s (1988) criteria, whereby values around |.10| indicate a small correlation, those around |.30| a moderate correlation, and those of |.50| or higher a strong correlation.

## Results

### Descriptive statistics for items

Table 2 shows descriptive statistics for PS-8 items, its two factors, and the other study variables. Some of the skewness and kurtosis coefficients for items of the PS-8 indicated deviation from the normal distribution, which justifies the use of the maximum likelihood and robust estimation methods for the CFA.

### Validity evidence based on internal structure

A CFA was conducted with the total sample to test the two-factor model of the PS-8. The fit indices showed a good fit, with values above .95 (CFI and NNFI) and below .06 (RMSEA). The fit of the model was then tested for men and women separately. Both models yielded satisfactory fit indices. Finally, the analysis of configural and metric invariance also indicated a good fit, insofar as there was a decrement of less than .01 in the CFI from the configural model to both the model with the factor loadings constrained and the model with the factor loadings and the covariance between factors constrained (Table 3). The values of standardized parameters for the total sample were all statistically significant (Table 4).

**Table 3:** Fit indices for the two-factor model of the PS-8

Model	*χ*^2^_S-B_	*df*	CFI	NNFI	RMSEA	Δ CFI
Total sample	33.05	19	.99	.99	0.04 [0.02, 0.07]	
Men	24.81	19	.99	.98	0.05 [0.00, 0.10]	
Women	36.72	19	.99	.98	0.06 [0.03, 0.09]	
Configural invariance	63.39	38	.99	.98	0.06 [0.03, 0.08]	
Equality constraints on factor loadings	73.31	44	.98	.98	0.06 [0.03, 0.08]	.003
Equality constraints on factor loadings and covariance	73.71	45	.98	.98	0.06 [0.03, 0.08]	.002

*Note. N* = 391; *men*, *n* = 132; women, *n* = 259; *χ*^2^_S-B_ = Satorra-Bentler chi-square; *df* = degrees of freedom; *CFI* = comparative fit index; *NNFI* = non-normed fit index; *RMSEA* = root mean square error of approximation with 90% confidence interval; *Δ CFI* = CFI configural invariance model–CFI constrained model

**Table 4:** Standardized factor loadings for the PS-8, corrected item-total correlations, and McDonald’s omega (*N* = 391)

PS-8 items	Factor loading	Item-total factor correlation
Communication disturbance		
1	.74	.61
2	.86	.72
3	.73	.61
4	.73	.59
Phone obsession		
5	.58	.48
6	.70	.54
7	.77	.60
8	.60	.45

### Reliability and item analysis

The corrected item-total correlations ranged from .45 to .72, well above the .30 threshold, thus indicating adequate item homogeneity. McDonald’s omega coefficient was .85 for communication disturbance and .76 for phone obsession, indicating satisfactory reliability for scores on both factors (Table 4).

### Validity evidence based on relationships with other variables

The correlation analysis indicated that scores on communication disturbance and phone obsession were positively related with time spent on the Internet on weekdays and at the weekend, time spent on social networking sites, number of social networks used, and scores on the measures of Internet addiction, problematic mobile phone use, Facebook intrusion, fear of missing out, and depression. The associations with time spent on social networking sites, Internet addiction, and problematic mobile phone use were strong, around |.50|, while the other correlations were weak or moderate (Table 5).

**Table 5:** Correlations of PS-8 scores with scores on the other variables

Variable	Communication disturbance	Phone obsession
Time spent on the Internet on weekdays	.21**	.24**
Time spent on the Internet at the weekend	.37**	.39**
Time spent on social networking sites	.43**	.42**
Number of social networks used	.22**	.31**
Internet addiction	.47**	.53**
Problematic mobile phone use	.57**	.59**
Facebook intrusion	.37**	.39**
Fear of missing out	.28**	.32**
Depression	.25**	.29**

Note. **p < .01; *p < .05

## Discussion

The aim of this study was to provide validity and reliability evidence to support the use of the PS-8 in the Portuguese population. To this end, we analyzed its internal structure through a CFA, examined measurement invariance across gender, the reliability of test scores, and item homogeneity, and obtained validity evidence based on relationships with other variables. We expected to obtain satisfactory fit indices for the correlated two-factor model for the PS-8 (communication disturbance and phone obsession), with measurement invariance across gender, satisfactory reliability coefficients for scores on both factors, adequate item homogeneity, and positive relationships of both factors with time spent on the Internet on weekdays and at the weekend, time spent on social networking sites, number of social networks used, Internet addiction, problematic mobile phone use, Facebook intrusion, fear of missing out, and depression.

As we expected, the CFA yielded adequate goodness-of-fit indices for a correlated two-factor model, with each factor comprising four items. The first factor, communication disturbance, refers to how individuals disturb their face-to-face communication by dealing with their mobile phones. The second factor, Phone Obsession, reflects the extent to which individuals need their mobile phone in environments lacking face-to-face communication. This structure is consistent with that reported in previous studies of both the 8-item and 10-item version of the Phubbing Scale (Błachnio et al., 2021; Błachnio & Przepiorka, 2019; Blanca & Bendayan, 2018; Gomes et al., 2021; Ivanova et al., 2020; Karadağ et al., 2015). This correlated two-factor model showed configural invariance across gender, indicating that items are indicators of the same latent factors with identical patterns of fixed and non-fixed parameters. This structure also supported metric invariance, indicating that the factor pattern coefficients are equal across groups and that items reflect the same latent construct for men and women. Metric invariance ensures that the strength of the relationships between items and their underlying factors are the same for both groups (Han et al., 2019). These results are also in line with the findings of Błachnio et al. (2021), who provided evidence for measurement invariance across gender in a sample collected from 20 different countries.

Regarding the item analysis, and according to our prior expectations, the correlations between item scores and the score on their respective factor indicated that items had satisfactory homogeneity. The reliability of factor scores was also satisfactory, with values of McDonald’s omega coefficient of .85 for communication disturbance and .76 for phone obsession, both above the .70 threshold.

Regarding validity evidence based on relationships with other variables, the analyses, as we expected, showed that scores on both factors were positively associated with time spent on the Internet on weekdays and at the weekend, time spent on social networking sites, number of social networks used, and scores on Internet addiction, problematic mobile phone use, and Facebook intrusion. These results confirm that the practice of phubbing is linked to various virtual addictions and the problematic use of technology, supporting the findings of other researchers. For example, previous studies have shown that phubbing is related to SMS and social network addiction (Al-Saggaf & O’Donnell, 2019), mobile phone addiction (Ivanova et al., 2020) and problematic social network use (Fang et al., 2020). In addition, Internet and mobile phone addictions have been found to be the most important predictors of phubbing (Davey et al., 2018; Yam & Kumcağız, 2020) which is consistent with our results here, insofar as the strongest correlation coefficients were those between the two phubbing factors and Internet addiction and problematic mobile phone use. Other studies have found that Facebook intrusion, defined as addiction and excessive attachment to Facebook (Elphinston & Noller, 2011), is also positively related to phubbing (Błachnio & Przepiorka, 2019; Blanca & Bendayan, 2018), which once again is in line with the present findings. Overall, these associations indicate that those individuals who more frequently ignore others by using their mobile phone during a face-to-face conversation, and who have a constant need for their phone in environments lacking face-to-face communication, tend to spend more time on the Internet and on social networking sites, use a greater number of social networking sites, and tend to show a stronger attachment to Facebook and more behavioral characteristics associated with addictive Internet and mobile phone use. These results confirm the addictive component of phubbing and support the view of Karadağ et al. (2015), who considered phubbing as the combined result of all virtual addictions.

The analyses also showed that scores on communication disturbance and phone obsession were positively associated with fear of missing out and depression. Fear of missing out refers to worries about being excluded from rewarding experiences that others are having (Przybylski et al., 2013). This fear involves not only a component of addiction to social networks, insofar as the individual wants to stay permanently connected, but also an anxiety component related to the possibility of exclusion from rewarding experiences. Notably, fear of missing out has been considered to bear similarities to an obsessive-compulsive disorder (Elhai et al., 2021; Przybylski et al., 2013). The association we observed between phubbing and fear of missing out is consistent with previous studies (Al-Saggaf & O’Donnell, 2019; Davey et al., 2018; Schneider & Hitzfeld, 2019; Yam & Kumcağız, 2020). As regards the association with depression, this indicates that individuals who practice phubbing tend to experience more depressive symptoms, highlighting the association between phubbing and mental health that has been reported previously (e.g., Davey et al., 2018; Ivanova et al., 2020). Given that phubbing is associated with feelings of exclusion and depression, some authors have proposed that those individuals who practice it might use their mobile phone as a way of coping with these feelings (Erzen et al., 2021; Smetaniuk, 2014). Others, by contrast, suggest that the practice of phubbing may lead to social exclusion and loneliness, leading in turn to depression (Ivanova et al., 2020).

This study has several limitations that need to be acknowledged. First, participants were not recruited by random sampling, which may restrict the generalizability of the findings. Second, data were obtained using self-report measures, which may be subject to response *bias*. Third, our use of a cross-sectional design means that no causal relationships can be inferred between the practice of phubbing and the other variables considered in the study. Despite these limitations, the current study provides further evidence regarding the psychometric properties of the PS-8, in this case in the Portuguese population.

## Conclusions

The PS-8 is a short and easy-to-administer scale that shows satisfactory goodness-of-fit indices supporting a correlated two-factor structure, which is invariant across gender. Item homogeneity and the reliability of factor scores were also satisfactory. Validity evidence was provided by associations between phubbing and several indicators of addictive use of the Internet, mobile phones, and social networking sites, as well as between phubbing and the fear of missing out and depression. Future studies should provide wider evidence about the relationships between PS-8 scores with other indicators of mental health and well-being such as anxiety, stress, life satisfaction, negative and positive affect, etc. Further longitudinal studies should also be included in order to analyze causal relationship and to identify potential mediators/moderators in this association.

## Data Availability

The datasets analyzed during the current study are available in the RIUMA repository, 10.24310/riuma.23226
